# St. Louis Encephalitis in Argentina: the First Case Reported in the Last Seventeen Years

**DOI:** 10.3201/eid0902.020301

**Published:** 2003-02

**Authors:** Lorena Spinsanti, Ana L. Basquiera, Sebastián Bulacio, Verónica Somale, Stefano C. H. Kim, Viviana Ré, Damián Rabbat, Abel Zárate, Juan C. Zlocowski, Carlos Quiroga Mayor, Marta Contigiani, Santiago Palacio

**Affiliations:** * Universidad Nacional de Córdoba, Córdoba, Argentina; † Hospital Privado Centro Médico de Córdoba, Córdoba, Argentina; ‡ Instituto de Radiología Conci-Carpinella, Córdoba, Argentina

**Keywords:** Encephalitis virus, St. Louis, seroconversion, substantia nigra, tremor, Argentina

**To the Editor**: St. Louis encephalitis is a mosquito-borne viral disease that affects humans. The causative agent, SLEV (formal name: *Saint Louis encephalitis virus*), is a member of the *Flaviviridae* family. Severity of the clinical syndromes increases with age, and persons >60 years old have the highest frequency of encephalitis. The primary transmission cycle involves wild passeiform, columbiform birds, and *Culex* *sp.* mosquitoes (*1*). In Argentina, an urban cycle may involve *Cx. quinquefasciatus*, which is a source of a viral isolate, and abundant birds (house sparrows, doves, or chickens) (*2*). The distribution of SLEV in Argentina is wide; seroprevalence ranges from 3% to 50% of the country’s population (*3*). Spinsanti et al. reported results of a serologic screening in persons ages 0–87 years who live in the city of Córdoba; antibodies were most frequently found in persons >60 years of age (*4*). However, cases of St. Louis encephalitis reported in Argentina are very rare. Two cases with serologic diagnosis were reported in 1964 and 1968, respectively (*2*). In 1971, two more cases were diagnosed on the basis of viral isolation (*5*). Finally, the last case reported was a patient with meningoencephalitis diagnosed in the province of Buenos Aires by hemagglutination inhibition assay (*6*). Herein, we report a case of Saint Louis encephalitis that occurred in the province of Córdoba, Argentina.

A 61-year-old man was admitted to the hospital in February 2002, complaining of headache, fever, and diplopia. He had been well until 3 months before admission, when ophthalmic herpes zoster was diagnosed. He underwent therapy with oral acyclovir and had a good clinical outcome. Ten days before admission, he developed unstable gait with misbalance and hand tremors, mainly at his left side. On admission, he had occipital headache, diplopia, and nausea and vomiting associated with high fever and chills. Somnolence appeared a few hours before the consultation.

The patient was a right-handed businessman, a native of Córdoba. He was married and had no risk factors for sexually transmitted diseases. He had not traveled inside or outside the country during the last year. He lived near a river with a high-density population of mosquitoes.

Vital signs on admission showed axillary temperature of 39°C, pulse of 90 beats per minute, respiratory frequency of 20 per minute, and blood pressure of 110/70 mmHg. Physical examination demonstrated a somnolent patient who was easily aroused and oriented. His speech was slurred. Results of a fundoscopic examination appeared normal. Results of a cranial-nerve examination showed horizontal left diplopia with left sixth nerve paresia. A resting, postural, and intentional hand tremor was evident. Motor strength was 5/5 throughout with normal bulk and tone, tendon reflexes, and coordination. Examination of sensitivity showed no abnormalities. A slight neck rigidity was detected.

Routine laboratory analysis was unremarkable, and results of serologic tests for coxsackie virus, echovirus, and HIV were negative. HIV-1 RNA by polymerase chain reaction (PCR) and p24 antigen were also negative. Cerebrospinal fluid study revealed a leukocyte count of 18/mm^3^ (80% lymphocytes), a glucose level of 48 mg/dL, and a protein level of 87 mg/dL. Cryptococcal antigen, antibodies for syphilis, Human herpesvirus 1 and 2, and PCR for varicella-zoster virus 1 and Human herpesvirus were also negative. Results of an electroencephalogram and a chest radiograph were normal. Therapy with intravenous acyclovir was initiated. A magnetic resonance imaging (MRI) scan of the brain showed a striking signal change on T2 in the substantia nigra of the midbrain, mainly at the right side.

The patient continued febrile, diplopia disappeared, and meningeal signs progressed with frank cervical stiffness, positive Kerning sign, and photophobia. Diffuse tremulousness and axial rigidity appeared. Upper extremities showed rigidity with cogwheel phenomenon. Conversely, lower extremities showed spasticity with bilateral Babinski sign. Tendon reflexes became enhanced. His gait showed retropulsion with wide base sustentation. Dysdiadochokinesia appeared. On the third day, a new lumbar puncture showed worse results: a leukocyte count of 210/mm^3^ (82% lymphocytes), a glucose level of 51 mg/dL, and a protein level of 106 mg/dL. Another electroencephalographic examination showed unspecific centroparietal disorganization with right side predominance. Intravenous acyclovir was stopped. On the 5th day, the patient began to recover; he was discharged on the 10th day. After 3 months of follow-up, only left arm rigidity and a left hand tremor persisted.

Acute- and convalescent-phase serum samples (taken 10 and 16 days after onset of illness, respectively) were sent to the Arbovirus and Arenavirus Disease Laboratory, Instituto de Virología, Córdoba. SLEV immunoglobulin (Ig) M antibodies were positive by indirect immunofluorescence assay (IFA). Seroconversion for IgG antibodies was demonstrated by IFA (*7*) and hemagglutination inhibition assay, with titers of 640 and 80 in the first sample and 2,560 and 320 in the second sample. These results were confirmed by neutralization test using the reduction of plates technique in Vero cells culture, as described (*8*). Eastern equine encephalomyelitis virus and Western equine encephalomyelitis viruses with known circulation in Argentina were included in the assay with negative results (*3*). An increase in antibodies titers between acute- (320) and convalescent-phase (1,280) samples was found only for SLEV. Among other flaviviruses, dengue, yellow fever, and Ilhéus circulate only in subtropical areas of Argentina (the province of Córdoba is not included in this area); only dengue virus was investigated (by neutralization test) because of a current epidemiologic surveillance program; results were negative. No evidence that West Nile virus is currently circulating or has entered Argentina was found, so we did not perform tests to detect it (*2*,*9*). Isolation of SLEV from the cerebrospinal fluid and blood was attempted in newborn mice and Vero cell cultures with negative results.

While the typical clinical manifestations of viral encephalitis (fever, headache, and altered level of consciousness) are indistinguishable from each other, tremor and other extrapyramidal signs are described in St. Louis encephalitis and Japanese encephalitis (*10*). The typical MRI finding of patients with St. Louis encephalitis is localized in the substantia nigra (*11*).

In summary, the occurrence of St. Louis encephalitis in a 61-year-old patient, after >10 years of no reports in Argentina, along with specific epidemiology, suggest that further studies are needed to assess the risk for human infection by SLEV in Argentina and the role of several mosquitoes species in its transmission.
